# A Scoping Review of Strategies for the Prevention of Hip Fracture in Elderly Nursing Home Residents

**DOI:** 10.1371/journal.pone.0009515

**Published:** 2010-03-03

**Authors:** Anna M. Sawka, Nofisat Ismaila, Ann Cranney, Lehana Thabane, Monika Kastner, Amiram Gafni, Linda J. Woodhouse, Richard Crilly, Angela M. Cheung, Jonathan D. Adachi, Robert G. Josse, Alexandra Papaioannou

**Affiliations:** 1 Division of Endocrinology, Department of Medicine, University Health Network, Toronto, Ontario, Canada; 2 Division of Endocrinology, Department of Medicine, University of Toronto, Toronto, Ontario, Canada; 3 Clinical Epidemiology Program, Ottawa Hospital Research Institute, Ottawa, Ontario, Canada; 4 Department of Clinical Epidemiology and Biostatistics, McMaster University, Hamilton, Ontario, Canada; 5 Department of Health Policy, Management, and Evaluation, University of Toronto, Toronto, Ontario, Canada; 6 Department of Medicine, McMaster University, Hamilton, Ontario, Canada; 7 School of Rehabilitation Sciences, McMaster University, Hamilton, Ontario, Canada; 8 Department of Surgery, Holland Orthopaedic & Arthritic Hospital of Sunnybrook Health Sciences Centre, Toronto, Ontario, Canada; 9 Division of Geriatrics, Department of Medicine, University of Western Ontario, London, Ontario, Canada; 10 Division of General Internal Medicine, Department of Medicine, University Health Network, Toronto, Ontario, Canada; 11 Division of Rheumatology, Department of Medicine, St. Joseph's Healthcare, Hamilton, Ontario, Canada; 12 Division of Rheumatology, Department of Medicine, McMaster University, Hamilton, Ontario, Canada; 13 Division of Endocrinology, Department of Medicine, St. Michael's Hospital, Toronto, Ontario, Canada; 14 Division of Geriatrics, Department of Medicine, Hamilton Health Sciences, Hamilton, Ontario, Canada; 15 Division of Geriatrics, Department of Medicine, McMaster University, Hamilton, Ontario, Canada; Swiss Paraplegic Research, Switzerland

## Abstract

**Background:**

Elderly nursing home residents are at increased risk of hip fracture; however, the efficacy of fracture prevention strategies in this population is unclear.

**Objective:**

We performed a scoping review of randomized controlled trials of interventions tested in the long-term care (LTC) setting, examining hip fracture outcomes.

**Methods:**

We searched for citations in 6 respective electronic searches, supplemented by hand searches. Two reviewers independently reviewed all citations and full-text papers; consensus was achieved on final inclusion. Data was abstracted in duplicate.

**Findings:**

We reviewed 22,349 abstracts or citations and 949 full-text papers. Data from 20 trials were included: 7 - vitamin D (n = 12,875 participants), 2 - sunlight exposure (n = 522), 1 - alendronate (n = 327), 1 - fluoride (n = 460), 4 – exercise or multimodal interventions (n = 8,165), and 5 - hip protectors (n = 2,594). Vitamin D, particularly vitamin D_3_ ≥800 IU orally daily, reduced hip fracture risk. Hip protectors reduced hip fractures in included studies, although a recent large study not meeting inclusion criteria was negative. Fluoride and sunlight exposure did not significantly reduce hip fractures. Falls were reduced in three studies of exercise or multimodal interventions, with one study suggesting reduced hip fractures in a secondary analysis. A staff education and risk assessment strategy did not significantly reduce falls or hip fractures. In a study underpowered for fracture outcomes, alendronate did not significantly reduce hip fractures in LTC.

**Conclusions:**

The intervention with the strongest evidence for reduction of hip fractures in LTC is Vitamin D supplementation; more research on other interventions is needed.

## Introduction

Hip fractures are not uncommon in the nursing home setting, with an estimated annual incidence rate of about 4% (range 2%–6%) [1]–[15]. This risk is two to eleven times that of community-dwelling elderly [5]–[9]. Within a year after sustaining a hip fracture, an elderly nursing home resident has a 40% risk of death [16] and a 6–12% risk of another hip fracture [16]; [17]. Most long-term care (LTC) facility residents surviving a hip fracture never regain pre-fracture functional status [18], and about two-thirds are left without independent mobility [19]; [20]. The estimated one-year healthcare cost of a hip fracture sustained in a LTC facility is about $33,000 (Canadian) [21]. Thus, hip fractures sustained in LTC facilities are an important source of mortality, morbidity, and healthcare expenditures. Interventions that may be effective in reducing the risk of hip fracture in community-dwelling elderly may not necessarily be effective in the LTC setting because of barriers such as resident comorbidities and concomitant prescribing contra-indications for osteoporosis drugs, frailty limiting exercise tolerance, or staff compliance with proper administration procedures (for drugs or devices).

Our objective was to conduct a scoping review (series of systematic reviews), to identify interventions proven in randomized controlled trials to reduce the risk of hip fracture in elderly nursing home residents. The summary of evidence in this review is intended to serve as a comprehensive resource for physicians, administrators, and health policy makers with an interest in LTC of the elderly. This review also highlights gaps in current knowledge and the need for more research in this area.

## Methods

### Inclusion Criteria for Studies

We included published randomized controlled trials or systematic reviews of randomized controlled trials of interventions intended to reduce the risk of hip fracture in the nursing home setting, as compared to placebo, sham intervention, usual care, or an alternative intervention. More than half of the study population was required to be comprised of elderly nursing home residents (or equivalent, defined as individuals of a median or mean age of ≥65 years of age, residing in an institution with nursing care available on-site 24 hours per day). Information on hip fracture outcomes was a requirement for inclusion (reported either in the primary publication or obtained from the author through correspondence). A follow-up period (mean or median) of at least 6 months was required. In the case of duplicate publications, only the largest published trial with longest follow-up period was included. For the systematic review of hip protectors, the inclusion criteria were the same as those described in recent systematic reviews by our group [22]; [23] as our most recent review [22] was updated herein. Ethical approval was not required for this study.

### Search Strategies

A review protocol was developed by experts in epidemiology/knowledge synthesis, biostatistics, health economics, endocrinology, geriatrics, and rheumatology (AS, LT, AG, AP, JA) (protocol available from the corresponding author upon request). There were 6 categories of interventions searched, including: a) vitamin D or calcium (or vitamin D analogues), b) non-hormonal pharmacologic therapies for osteoporosis, c) hormonal therapies (or hormone analogues), d) oral or parenteral alternative medicines, e) exercise, behavioral interventions, physiotherapy, education, or multimodal interventions (including fall prevention strategies), and f) hip protectors. Electronic search strategies were directed by a librarian experienced in systematic reviews. There were no language restrictions. Additional articles were identified through reference lists or discussions with experts. The details of the databases searched are listed in the Appendix S1.

### Selection of Studies for Inclusion

Two reviewers independently scanned all abstracts and citations and decided on relevance to the review. Any abstracts or citations deemed potentially relevant by either reviewer were reviewed in full-text form by two independent reviewers. Full-text papers were included in this review only if consensus was achieved between reviewers.

### Validity Assessment

Two reviewers independently assessed methodologic quality of included studies. Reviewers were not masked to the study authors nor journals of publication. Key features in methodologic quality assessment included a description of the level of randomization (individual or cluster), the method of randomization, the utilization of placebo or sham interventions, losses to follow-up, compliance rates, and reporting of adverse events. In addition, for cluster randomized trials, we examined whether the intra-cluster correlation coefficient (or variance inflation factor attributed to clustering) was reported and if the analysis was adjusted for cluster randomization. The results of the validity assessment are summarized in the Results under Study Characteristics.

### Data Abstraction

All data were abstracted independently by two reviewers. A data abstraction form including study characteristics and fracture outcomes was utilized (sample data abstraction form available from the corresponding author upon request). All data were reviewed by AMS and any discrepancies were resolved among reviewers. For the intervention of exercise or behavioural, or multi-modal interventions, a supplemental data abstraction form was provided to summarize falls outcomes as these were deemed relevant for this particular set of interventions.

### Statistical Analyses

Separate statistical analyses were planned for each of the 6 respective categories of interventions. A previously described Bayesian random effects meta-analysis for pooling of intention-to-treat data from individually or cluster randomized trials in the nursing home setting [22] was used (Winbugs 1.4.1, MRC Biostatistics Unit, Cambridge, UK) (random seed number 314159). We used non-informative priors in Bayesian models, as previously published [22]. For the oral vitamin D meta-analyses, we performed 10,000 simulations, with Gibbs sampling of results for posterior distributions started at 1,000 (sampling every fifth value); three chains were run simultaneously. For the hip protector meta-analysis, we performed 10,000 simulations, with Gibbs sampling of results for posterior distributions started at 1,000 (sampling every fifth value); three chains were run simultaneously. For pooled cluster-randomized trials, an intra-cluster correlation coefficient (ICC) of 0.0247 was imputed, if not reported [22]. This ICC was derived from a comprehensive sensitivity analysis as published previously [22]. We calculated the pooled odds ratio (OR) of hip fracture in the treatment group relative to the control group, with 95% credibility interval (CRI) [22]. A priori planned subgroup analyses for oral vitamin D included separate analyses for any form of vitamin D, vitamin D_2_, vitamin D_3_, and vitamin D_3_ ≥800 IU daily (or equivalent), all based on findings of a prior review [24]. If a Bayesian meta-analysis could not be performed, due to insufficient data or non-convergence of posteriors, a classical random effects meta-analysis was performed, estimating the odds ratio of hip fracture in the treatment group with 95% confidence intervals (CI) (Comprehensive Meta-Analysis software, Biostat, Englewood, NJ, USA). Although the frequentist approach to meta-analysis is much more common and popular in medical research, one of its limitations is a lack of flexibility of modeling, particularly relevant here when pooling data from cluster and individually randomized trials. Between-study heterogeneity was calculated on the log scale from posterior distribution of the variance parameter of the Normal distribution of the log OR estimates for Bayesian meta-analyses [22]. Between-study heterogeneity was assessed by calculation of the I^2^ and Q-values for the frequentist meta-analyses. For individually randomized trials which could not be pooled, and for which no statistical analysis was reported in the primary paper, we calculated the odds ratio of hip fracture with 95% CI using an intention-to-treat approach (CIA software, London, UK). The 25-hydroxy-vitamin D measurements were converted from ng/ml to nmol/L by multiplying by 2.496 [24]. Given the complexity of this scoping review, in that multiple reviewers in multiple institutions, were involved in multiple updates of multiple reviews, reviewer agreement statistics were not formally calculated. More details on the analyses are available from the corresponding author upon request. Other details relating to the systematic review methodology are otherwise summarized in Checklist S1 (Prisma Checklist).

## Results and Discussion

### Study Characteristics

The numbers of abstracts/citations and full-text papers reviewed in each category of review are summarized in Table 1 (summary of trial flow). Within 6 respective searches (by category of intervention), there was a total of 22,349 abstracts or citations and 949 full-text papers reviewed in duplicate. Data from 20 randomized controlled trials were included in the final scoping review, including: 7 trials on oral vitamin D (with or without calcium) (total of 12,875 participants) [[25];[27]–[32], with supplemental information on reference 25 from reference 26], 2 trials on sun exposure (as a form of vitamin D treatment) (total of 522 participants) [33]; [34], 1 trial on alendronate (n = 327 participants) [35], 1 trial on fluoride (including a total of 460 participants) [36], 4 trials on exercise/behavioral, or multimodal interventions (total of 8,165 participants) [37]–[40], and 5 trials on hip protectors (total of 2,594 participants) [41]–[45], of which four [41]–[44] were previously summarized in a prior systematic review by our group [22]) (Table 2). A recent hip protector trial by Kiel et al. (2007) [46] was excluded from our review since participants who dropped out or died were substituted with nonrandomized individuals, violating an a priori exclusion criterion for this review [22]; [23].

**Table 1:** Summary of the citations and full-text papers reviewed.

Review category	Citations and abstracts reviewed (Electronic search source)	Full-text papers reviewed	Included papers	Excluded papers	Reasons for exclusion of full-text papers reviewed							
					Not population of interest	Narrative review or commentary	Norandomization	Hip fracture outcomes not reported	Follow up <6 months	Overlap of included data	Not intervention of interest	Other reason
Vitamin D or calcium	3069 (3062)	163	9	154	70	55	8	10	2	7	2	0
Pharmacologic therapies	3107 (3091)	267	1	266	210	39	6	2	1	5	3	0
Hormonal therapies	3508 (3507)	264	0	264	113	115	31	1	0	3	1	0
Alternative medicines	6812 (6810)	157	1	156	70	28	43	15	0	0	0	0
Exercise, behavioral or multimodal interventions	5672 (5668)	81	4	77	51	1	10	7	1	1	6	0
Hip protectors	181 (180)	17	2	15	0	7	3	0	0	3	1	1*

* For the update of a prior systematic review of hip protectors, an a priori established exclusion criterion was the substitution of participants who dropped out or died with non-randomized participants.

**Table 2:** Description of the included studies and pooled analyses.

Review category	Author and year (Ref)*	Intervention	Study population (Sex)	Number participants randomized (Institutions)	Follow-up duration (Months)	Number of Hip Fractures in Treatment (T) and Control (C) groups (Percentage)	Pooled Odds Ratio (OR) of Hip Fracture in Intervention Group Compared to Controls † (Number of participants and trials)
**Vitamin D or calcium**	Chapuy 1994 [25]	Vitamin D_3_ 800 IU+1.2 g calcium daily	All from nursing homes (All female)	3270 (180)	36	T: 138/1634 (8.4%) C: 184/1636 (11.2%)	Oral vitamin D: Pooled OR 0.86 (95% credibility interval, 0.74, 0.98) (Pooled n = 12,875 in 7 trials)
	Chapuy 2002 [27]	Vitamin D_3_ 800 IU+1.2 g calcium daily	All from nursing homes (All female)	583 (55)	24	T: 27/393 (6.9%) C: 21/190 (11.1%)	
	Flicker 2005 [28]	Vitamin D_2_ 10,000 IU/week or 1,000 IU per day+0.6 g calcium daily	Nursing home and other institutions (Male and female)	625 (149)	24	T: 3/313 (1.0%) C: 7/312 (2.2%)	
	Law 2006 [29]	Vitamin D_2_ 2.5 mg every 3 months (equivalent to 1,100 IU/day)	Nursing home and other institutions (Male and female)	3717 (118)	10 (Median and mean)	T: 24/1762 (1.4%) C: 20/1955 (1.0%)	
	Lyons 2007 [30]	Vitamin D_2_ 2.5 mg three times per year (About 833 IU/day)	Nursing home and other institutions (Male and female)	3440 (314)	36	T: 127/1725 (7.4%)‡ C: 126/1715 (7.3%)‡	
	Meyer 2002 [31]	Vitamin D_3_ 400 IU daily in 5 ml cod liver oil	All from nursing homes (Male and female)	1144 (51)	24	T: 50/569 (8.8%) C: 47/575 (8.2%)	
	Sato 2005 [32]	Vitamin D_2_ 1,100 IU daily	Chronic geriatric hospitalization (Females, post-stroke with hemiplegia)	96 (1)	24	T: 0/48 (0%) C: 4/48 (8.3%)	
	Sato 2005 (Sunlight) [33]	Outdoor sunlight exposure in clear weather for 15 minutes daily 1.2 g calcium daily	Chronic geriatric hospitalization, Alzheimer's Disease (all females)	264 (1)	12	T: 2/132 (1.5%) C: 9/132 (6.8%)	Sunlight exposure: Pooled OR 0.43 (95% confidence interval 0.10, 1.83) (n = 522 in 2 trials)λ
	Sato 2003 (Sunlight) [34]	Outdoor sunlight exposure in clear weather for 15 minutes daily	Chronic geriatric hospitalization, post-stroke hemiplegia (Males and females)	258 (1)	12	T: 1/129 (0.8%) C: 6/129 (4.7%)	
**Drug therapies**	Greenspan 2002 [35]	Alendronate 10mg orally daily+Vitamin D 400 IU daily (type not specified) +Calcium carbonate (OsCal 500, if dietary calcium <1500 mg/day)	Nursing home and other institutions (All female)	327 (25)	24	T: 2/163 (1.2%)ϕ C: 4/164 (2.4%)ϕ	OR 0.50 (95% confidence interval 0.09, 2.75) λϕ (n = 327 in 1 trial)
**Alternative medicines**	Inkovaara 1975 [36]	Sodium monofluorophosphate (25 mg of fluorine daily for first 5 months, then 25mg twice a week)	Municipal home for the aged (Males and females)	460 (1)	8	T: 8/237 (3.4%) C: 5/223 (2.2%) (Femoral)	OR 1.52 (95% confidence interval 0.49, 4.73) λϕ (n = 460 in 1 trial)
**Exercise, behavioral or multimodal**	Becker 2002 [37]	Multimodal intervention: Staff and resident education, balance and resistance training, hip protectors	All in nursing homes (Males and females)	981 (6)	12	T: 17/509 (3.3%) C: 15/472 (3.2%)	Relative Risk 1.11 (95% confidence interval 0.49, 2.51) λ∂ (Adjusted for cluster randomization) (n = 981 in 1 trial)
	Cox 2003 [38]	Multimodal intervention including staff education and individual risk assessment with feedback and advice to physicians	Nursing home and other institutions (Male and female)	6229 (58 PCO clusters)	12	T: Not reported/3476 C: Not reported/2753	Relative Risk 0.86 (95% confidence interval 0.63, 1.18) λ∂£ (Adjusted for cluster randomization) (n = 5637 residents in 1 trial)
	Jensen 2002 [39]	Multimodal intervention: Staff education, environmental modification, exercise program, drug modification (for falls prevention), select specialist referral, hip protectors	All in nursing homes (Males and females)	402 (9)	11 week intervention with 34 week follow-up	T: 3/194 (1.5%) C: 12/208 (5.8%)	Unadjusted OR 0.25 (95% confidence interval 0.05, 1.13)£ λ∂ OR Adjusted for Baseline Variables 0.23 (95% confidence interval 0.06, 0.94)£ ∂μ (All adjusted for cluster randomization) (n = 384 in 1 trial)
	Sakamoto 2006 [40]	Exercise: Daily supervised uni-pedal standing balance exercise	Nursing home and other institutions (Male and female)	553 (32)	6	T: 1/337 (3.0%) C: 1/216 (4.6%)	Not significant (p>0.999) λ∂£ (n = 527 in 1 trial)
**Hip protectors**	Sawka 2007 (Systematic review, data from 4 trials) [22] (Ekman 1997, Harada 2001, Jantti 1998, Meyer 2003)	Hip protectors (Shields on both hips)	All nursing home residents (Harada - all female, others - male and female)	1,922 (Ekman – 4, Harada – 6, Jantti - 1, Meyer 42)	Range 11–15	Ekman: T: 4/302 (1.3%) C: 17/442 (3.8%) Harada: T: 1/88 (1.1%) C: 8/76 (10.5%) Jantti: T: 1/36 (2.8%) C: 7/36 (19.4%) Meyer: T: 21/459 (4.6%) C: 42/483 (8.1%)	Hip Protectors Pooled OR 0.40 (95% credibility interval 0.27, 0.56) (Pooled n = 2,594 individuals in 5 trials)
	Koike 2009 [45]	Hip protectors (shields on both hips) and leaflet on fracture prevention	All nursing home residents (or equivalent)‡ (All female)	672	12–26σ	T: 9/345 (5.5%) C: 39/327 (11.9%)	

*Ref is the reference number listed at the end of this paper.

†Intention to treat analysis unless otherwise indicated.

‡The primary author confirmed through e-mail correspondence that all residents had onsite 24-hour per day nursing care available.

σTime from allocation to treatment to end of follow-up period (allocation in January, 2004 and March, 2005 to end of follow-up in March, 2006).

λ A Bayesian random effects meta-analysis could not be performed for this data.

ϕCalculated.

∂Results reported in the primary paper.

£The treatment effect reported by the primary author is not an intention to treat analysis as the number randomized is higher than the number for which the statistical test was performed.

μThe baseline variables adjusted for in this model included: Mini-Mental Status Examination score, Barthel index score, physical restraints and delirium, sex and history of falls, and age.

### Assessment of the Methodologic Quality of Included Studies

A summary of the key features in the assessment of methodologic quality of included studies is shown in Table 3. Central computerized randomization was variably performed in included trials. Placebo controls were used in most trials of oral vitamin D (with the exception of the study by Law [29]), as well as the trials of alendronate [35] and fluoride [36]. Placebo or sham interventions were not used in any of the trials of exercise/behavioral, or multi-faceted interventions. With the exception of three trials of oral vitamin D (Chapuy 1994 [25], Chapuy 2002 [27], and Lyons 2007 [30]) in which compliance rates exceeded 80%, compliance was lower or not reported for the rest of the intervention studies (Table 3). Compliance was lower than 80% in all of the hip protector trials (Table 3). Fewer than half of studies in this review reported losses to follow-up of less than 10% (excluding deaths) and fewer than half reported the presence or absence of adverse events (Table 3). Most cluster randomized studies reported the unit of randomization [29;37]–[39;41;44;45] and adjusted the treatment effect analysis for cluster randomization [29;37]–[39;44]; whereas the ICC was formally reported only in the multimodal intervention trial by Cox et al. [38].

**Table 3:** Assessment of the methodologic quality of the included studies.

Review category	First author and year (Ref)*	Level of randomization	Central computerized randomization	Use of placebo or sham in control group	Losses to follow-up <10% (Excluding deaths)	Compliance with treatment intervention >80%	Reported adverse events
**Vitamin D or calcium**	Chapuy 1994† [25]	Individual	Not reported	Double placebo (for vitamin D and calcium)	Yes	Yes at 18 months	Yes
	Chapuy 2002 [27]	Individual	Not reported	Double placebo (for vitamin D and calcium)	No	Yes	Yes
	Flicker 2005 [28]	Individual	Yes	Placebo (weekly or daily) +0.6 g calcium daily	No	No	Yes (No adverse events occurred)
	Law 2006 [29]	Cluster (30-bedded residential unit or small facility without units)	Yes	No (Usual care)	Yes	Not reported (2% stopped treatment)	Yes
	Lyons 2007 [30]	Individual	Yes	Placebo	Yes	Yes	No
	Meyer 2002 [31]	Individual	No (Allocated by day of birth)	Placebo	No	Unclear (2% stopped treatment)	No
	Sato 2005 [32]	Individual	Yes	Placebo	Unclear	Not reported	No
	Sato 2005 (Sunlight) [33]	Individual	Yes	No (Usual care+1.2g elemental calcium daily)	Yes	Not reported	No
	Sato 2003 (Sunlight) [34]	Individual	Yes	No (Usual care)	No	Not reported	Yes (No adverse events occurred)
**Drug therapies**	Greenspan 2002 [35]	Individual	Yes	Placebo+Vitamin D 400 IU daily (type not specified) +Calcium carbonate (OsCal 500, if dietary calcium <1500 mg/day)	Not reported	Not reported	Yes
**Alternative medicines**	Inkovaara 1975 [36]	Individual	No (Allocated by year of birth – odd or even number)	Matched placebo (30 mg sodium bicarbonate)	No	Not reported	Yes
**Exercise, behavioral or multimodal**	Becker 2002 [37]	Cluster (Nursing home)	No (Allocated by sealed envelopes)	No (Usual care)	Yes	No	No
	Cox 2003 [38]	Cluster (Primary Care Organizations [PCO] representing multiple nursing homes)	Yes	No (Usual care)	Yes for individuals	Not clearly reported	No
	Jensen 2002 [39]	Cluster (Nursing home)	No (Allocated by sealed envelopes)	No (Usual care)	No	Not clearly reported for all components of intervention	No
	Sakamoto 2006 [40]	Individual	No (Allocated by random number table)	No (Usual care)	Yes	Not clearly reported	No
**Hip protectors**	Sawka 2007 (Systematic review, data from 4 trials)‡ [22]	Jantti – Individual Others - Cluster	(Ekman 1997, Harada 2001, Jantti 1998, Meyer 2003) Yes - Meyer study, not others	No (Usual care)	No	No (Unclear for Meyer 2003λ)	No
	Koike 2009 [45]	Cluster	No (Random number table)	No (Usual care and a leaflet on fracture prevention)	Yes	No (79.7%)	Yes

*Ref is the reference number listed at the end of this paper.

†Some data reported in a prior paper by Chapuy et al. in 1992 on the same study population [reference 26].

‡Some data reported in a prior paper by Sawka et al. in 2005 [reference 23] or the primary references.

λIn the hip protector study by Meyer et al. [reference 44], the primary author relayed through e-mail correspondence that hip protector compliance was not measured, but a “worst-case” estimate of 34% for hip protector use in the treatment group was reported.

### Vitamin D or Calcium

We included 7 randomized controlled trials on oral vitamin D, including 3 trials utilizing vitamin D_3_ [25]; [27]; [31], and 4 trials utilizing vitamin D_2_ [28]–[30;32]. In two vitamin D_3_ trials from Chapuy et al [25]; [27], the control group received double placebo instead of calcium and vitamin D, respectively. In the other vitamin D studies, a specific placebo was not substituted for calcium supplements (Table 3). However, none of the studies in the long-term care setting was found to examine the independent effect of calcium supplementation on hip fracture rates. In pooling data from trials of oral vitamin D compared to placebo or usual care (12,875 individuals) [25;27]–[32], the odds ratio (OR) for hip fracture in the vitamin D-treated group was 0.86 (95% credibility interval [CRI], 0.74, 0.98) (between-study heterogeneity on a log scale, mean 1.40×10^−4^, 95% CRI 1.40×10^−7^, 6.77×10^−4^) (Table 2). The pooled odds ratios (with 95% CRI) for hip fracture in the treatment group according to type and dose of vitamin D are as follows: vitamin D_3_ (any dosage) - OR 0.78 (0.63, 0.93) (data from 3 trials [25]; [27]; [31], n = 4997), vitamin D_3_ at a dosage of ≥800 IU/day (with 1.2 g elemental calcium daily) – OR 0.71 (0.55, 0.87) (data from 2 trials [25]; [27], n = 3853), vitamin D_2_ – OR 0.99 (0.79, 1.22) (data from 4 trials [28]–[30;32], n = 7878). There was only one trial in which a dosage of 400 IU daily of vitamin D_3_ was utilized, and the relative risk of hip fracture in the treatment group was reported to be 1.09 (95% confidence interval, 0.63, 1.63) [31]. Adverse events were very uncommon in the trials of oral vitamin D, with the following incidence rates of hypercalcemia reported: 0.1% in the trial by Chapuy et al (1994) [25], 0.8% in the trial by Chapuy et al (2002) [27], and 0.1% in the trial by Law et al. [29]; whereas there were no adverse events in the trial by Flicker et al. [28]. The incidence of gastrointestinal side effects such as nausea, diarrhea, or epigastric pain was similar between treatment and control groups in the two trials by Chapuy et al. [25]; [27]. The mean baseline 25-hydroxyvitamin D blood levels of participants were reported to be ≤40 nmol/L in the following trials of oral vitamin D: Chapuy 1994 [25], Chapuy 2002 [27], and Sato 2005 [32]. The mean baseline hydroxyl-vitamin D blood measurements were >40 nmol/L in the studies by Lyons et al. [30] and Meyer et al. [31] and in the control group studied by Law et al. [29]; these data were not reported by Flicker et al. [28].

In a pooled analysis of intention-to-treat data from two trials in which 15 minutes of daily sunlight exposure (on clear days) was used as a form of vitamin D treatment [33]; [34], the odds ratio of hip fracture in the treatment group was 0.43 (95% confidence intervals, 0.10, 1.83, n = 522) (between-study heterogeneity I^2^ value of 34.5, Q-value 1.53 [1 degree of freedom], p = 0.217) A classical random effects meta-analysis was used since the Bayesian random effects model could not achieve convergence using this small dataset of small trials. No adverse events as a consequence of sunlight exposure were reported by Sato et al. in one of these trials [34]. The mean baseline 25-hydroxyvitamin D levels were ≤40 nmol/L in the two trials testing sunlight exposure [33]; [34].

### Pharmacologic and Alternative Therapies

Greenspan et al examined the use of oral alendronate 10 mg daily compared to placebo in one randomized controlled trial including 327 institutionalized elderly in 25 institutions [35]. In this study, which was not powered to detect differences in fracture outcomes, the OR of hip fracture in the treatment group was calculated to be 0.50 (95% CI 0.09, 2.75). Adverse events were not significantly increased in the alendronate-treated group compared to the controls in this study [35].

In one trial comparing oral fluoride to placebo [36], hip fractures were not significantly reduced in the treatment group (OR 1.52, 95% CI 0.49, 4.73). Moreover, this study was terminated early due to excessive rates of abdominal discomfort, weight loss, hospitalization, and spontaneous fractures experienced in the fluoride group [36].

### Exercise, Behavioral, or Multimodal Interventions

One exercise study [40] and 3 trials of multimodal interventions [37]–[39] were included in this review (Table 2). In the one study comprised of strictly an exercise intervention, Sakamoto et al. asked participants in the intervention arm, to stand on their right leg for one minute and then their left leg for another minute, for a total of 2 minutes, three times a day (with eyes open); a control group was observed without any exercise intervention in the same time period [40]. In the study by Sakamoto et al, the unipedal standing exercise program was reported to reduce falls (although one treatment group participant who fell frequently was excluded from the analysis, not intention-to-treat analysis) (p<0.001); however the risk of hip fracture was not significantly reduced over six months (n = 553 participants randomized) [40]. Becker et al. tested a multimodal intervention comprised of any combination of the following components over 12 months (with choice of resident-specific interventions dependent on resident preference): a) staff training in a 60-minute course with written information on falls, b) written information on fall prevention for all residents, with a personal consultation on fall prevention offered by a nurse or exercise instructor to all residents who were not chair- or bed-bound, c) an environmental hazard check of the facility with discussion of the results with staff and administrators, d) balance exercises and progressive resistance training with ankle weights and dumbbells (20 minutes of balance exercise in a standing position and walking if possible with nine different standardized progressive resistance training exercises for a maximum of 10 repetitions of resistance exercises in two sets, including all major muscle groups), and e) hip protectors offered to residents who could stand (assisted or unassisted) or who could rise from a chair unattended (5 pairs of either Safety Pants, Raunomo Oy, Tampere, Finland or Safehip, Tytex, Ikast, Denmark) [37]. Although falls were significantly reduced in the study by Becker et al (relative risk [RR] 0.55, 95% confidence interval [CI] 0.41, 0.73), hip fractures were not significantly reduced (RR 1.11, 95% CI 0.49, 2.51) (n = 981 participants randomized) [37]. In another study of a multi-modal general and resident-specific intervention, Jensen et al studied the following strategy: a) a 4-hour educational session on falls for staff, b) environmental modification by staff and study physiotherapists (including removal of loose carpets, provision of grip bars, new beds, new firm mattresses, furniture changes, and improved lighting), c) supervised resident-specific exercise training to improve physical function, focusing on strength, balance, gait, and safe transfer, d) supply and repair of mobility-related aids by physiotherapists, e) medication adjustments intended to reduce fall risk, f) provision of hip protectors to residents who were considered particularly prone to sustaining a fall-related hip fracture, g) post-fall problem-solving conferences, and h) staff guidance by researchers with ongoing discussions about safety issue of fall-prone residents [39]. In the study by Jensen et al, all residents were screened for their risk of falling (interviewed and assessed by physiotherapists at baseline); individuals in the control arm received usual care [39]. Jensen et al reported a significantly reduced risk of falls in the treatment group compared to controls (risk ratio 0.78, 95% CI 0.64, 0.96) and that the risk of hip fracture was significantly reduced in a secondary analysis adjusting for baseline variables (relative risk 0.23, 95% CI 0.06, 0.94, n = 384, not intention-to-treat analysis). In a third trial, Cox et al. studied the following multimodal intervention over 12 months: a) a half-day central training session with written materials for staff focusing on bone health and osteoporosis, falls and fall prevention, risk factors for falls and fractures, and risk assessment tools for fracture (the Black fracture risk assessment tool and the STRATIFY fall risk assessment tool), b) care home staff assessing individual risk of fracture and falls using the Black and STRATIFY tools with results sent to specialist osteoporosis nurses, and c) individual assessment results and treatment recommendations sent from specialized osteoporosis nurses to treating physicians [38]. In the study by Cox et al., falls, total fractures, or hip fractures were not significantly reduced by the educational and risk assessment/recommendation strategy, even though rates of prescription of bisphosphonates and calcium/vitamin D rose (n = 6229 participants randomized) [38].

### Hip Protectors

As part of this review, we updated a prior systematic review from our group [22]. Our original review included data from 4 trials, including those from Ekman 1997 [41], Harada 2001 [42], Jantti 1998 [43], Meyer 2003 [44]. In this update, an additional study was included from Koike et al. 2009 [45]. All of these trials tested 2-sided hard shell hip protectors, and the Safehip brand was used by Harada [42], Meyer (2003), and Koike (2009) [45]. In the newest study by Koike et al., female residents were eligible to participate in the trial if they were aged ≥65 years and had at least one of the following risk factors for fracture: history of prior fracture, low body-mass index, family or individual history of hip fracture, frequent faller status, current smoker, or other frail residents [45]. Four of the hip protector trials [41]; [42]; [44]; [45] included in this review were cluster randomized, with the unit of randomization being the entire LTC facility in three trials [41]; [44]; [45] or a room (usually containing 4 beds) in one study [42]. The incidence of hip fracture in the control groups of included studies ranged from 3.8% to 19.4% over the course of the studies (range of respective trial durations 11 to 26 months) ([41]–[45], Table 2). In pooling data from the 5 hip protector trials, the odds ratio of hip fracture in the treatment group was 0.40 (95% credibility interval 0.27, 0.56) (n = 2,594 individuals, Table 1) (between-study heterogeneity on a log scale, mean 1.70×10^−4^, 95% CRI 2.01×10^−7^, 8.60×10^−4^). The adherence rate for the hip protector trials was as follows: 44% at 11 months in the study by Ekman [41], 70% at 12 months in the study by Harada [42], 68% at 12 months in the study by Jantti [43], 79.7% throughout the study period (up to 26 months) in the study by Koike et al. 2009 [45], and 34% (“worst case estimate”) in the study by Meyer [44].

It is important to acknowledge the findings of a recent trial by Kiel et al. (2007), in which institutions were cluster randomized to either a right or left-sided hip protector as this study reported findings that are contradictory to our pooled analysis [46]. The study by Kiel et al. was excluded from our review since participants who dropped out or died were substituted with nonrandomized participants, violating an a priori exclusion criterion established in our prior review on this topic (and potentially resulting in bias, if participants from one group were preferentially replaced) [22]; [23]. However, in the study by Kiel et al. [46], the incidence of hip fracture on the protected side (3.1%, 95% CI 1.8, 4.4%) was not significantly different compared to the unprotected side (2.5%, 95% CI 1.3, 3.7%) (p = 0.70) (n = 1042 protected and 1042 unprotected hips followed for 20 months). This study was terminated early because of futility [46].

### Summary and Discussion

In summary, we have reviewed data from randomized controlled trials of interventions for the reduction of risk of hip fracture in elderly LTC facility residents. Using a Bayesian meta-analysis, we found evidence that supplementation with Vitamin D, particularly Vitamin D_3_ ≥800 IU daily, reduces the risk of hip fracture in elderly nursing home residents. These results are in keeping with prior results of meta-analyses by Bischoff-Ferrari et al., particularly for doses of vitamin D_3_ exceeding 400 IU per day, where data were pooled from institutionalized and community populations using a non-Bayesian analytic technique [24]; [47]. Neither sunlight exposure nor fluoride treatment was found to significantly reduce hip fractures. In one trial examining the use of oral alendronate in institutionalized elderly, hip fractures were not significantly reduced; however the trial was not adequately powered to detect fracture outcome differences (n = 327) [35]. It is relevant to note that in a recent Cochrane systematic review and meta-analysis, alendronate was shown to reduce the risk of hip fracture in high risk secondary prevention populations [48], upon pooling data on institutionalized elderly [35] with community populations. Thus, it could be extrapolated that potent amino-bisphoshonates such as alendronate may reduce hip fracture when used in secondary prevention in nursing home residents who do not have prescribing contra-indications and who are able to tolerate the drug upon proper administration. In the alendronate trial performed in a LTC population, adverse events were not suffered to a greater extent in the treatment group compared to controls [35], albeit relatively small sample size may have been a limiting factor in detecting this outcome. In a trial of a unipedal standing exercise program, Sakamoto et al. reported a reduced risk of falls in the LTC setting but no significant effect on hip fractures [40]. Falls were also reduced in two studies of multimodal interventions [37]; [39], with one study of a multi-modal intervention by Jensen et al. suggesting a reduction in hip fractures in one of several secondary analyses [39]. Some important differences between the multimodal intervention offered by Jensen et al. [39], in which hip fractures were reduced, compared to the intervention offered Becker et al. [37], in which hip fractures were not reduced, included: more intensive staff training, more ongoing support on falls prevention, supply and repair of mobility aids, and medication adjustments, in the former study [39]. Of note, in a study by Cox et al, a staff education and risk assessment strategy did not significantly reduce falls or hip fractures in LTC facilities [40]. One of the main differences between the program offered by Cox et al. [38] compared to the interventions provided by Becker et al. [37] and Jensen et al. [39] is the performance of environmental hazard checks in facilities as well as direct offering of exercise programs or hip protectors to residents in the latter studies. In this review, we also found some evidence that hip protectors may reduce the risk of hip fracture in institutionalized elderly in a pooled analysis of 2-sided devices. However, a recent large trial of 1-sided hip protectors was negative [46], igniting some controversy about this intervention. As acknowledged by the authors of this study, “the use of a single pad is not analogous to pad use in the real world and may have caused unanticipated changes in behavior” [49]. Continued debate and uncertainty about the efficacy of hip protectors is expected, given the heterogeneity of findings between studies of 2-sided and 1-sided devices. However, given that 2-sided devices are the norm used in clinical practice, and that pooled analyses or randomized controlled trials of this intervention have shown reduction of hip fractures, it may not be appropriate to discount the potential benefit of this intervention in the LTC setting.

This scoping review is subject to several limitations, including a relative paucity of large trials such as those of pharmacologic therapies, the relatively small size of some of the included studies, the imputation of intra-cluster correlation coefficients for pooled cluster randomized studies not reporting this value, the inherent methodologic limitations of many of the primary studies (such as poor reporting of compliance rates, a lack of placebo or sham interventions for trials of some interventions, and the lack of reporting of intention-to-treat analyses for some studies), the possibility of reporting bias, and the potential for publication bias (as only published studies were included). We did not specifically assess reporting or publication bias in this review, given the relatively small number of studies included in each category. The strengths of this review include the relatively broad scope of interventions examined, the use of systematic search strategies, duplicate reviews and duplicate abstraction of data, and the use of a Bayesian meta-analysis model designed for pooling of data from individually and cluster-randomized trials, and the examination of a clinically important outcome.

In conclusion, we would recommend routine supplementation with Vitamin D_3_ ≥800 IU daily for elderly nursing home residents, in considering the potential benefit in reduction of hip fractures, general ease of administration, and low incidence of side effects of this intervention. Multimodal strategies, including staff and resident education on falls and fractures, environmental modification, exercise training, and offering of hip protectors, as those described in the trial by Jensen et al. [39] appear to be beneficial in reducing falls, which is a desirable outcome in itself and may warrant implementation, if feasible and affordable for a facility. However, larger studies are required to confirm whether such multimodal interventions reduce hip fractures in LTC. Decision making about pharmacologic treatment with potent bisphosphonates or application of hip protectors is complex, given that the former has not been proven to reduce the risk of hip fractures in the nursing home setting (with evidence of efficacy of hip fracture prevention largely founded in larger community-based studies], and some conflicting findings in the medical literature for the latter intervention. In considering the limitations of the best available evidence, it may be reasonable to reserve pharmacologic therapy or hip protectors for nursing home residents at highest risk of hip fracture, such as residents with prior fragility fracture or multiple risk factors (especially if any vitamin D insufficiency or deficiency has first been treated]. Careful consideration must be given to any contraindications to bisphosphonate therapy or the feasibility of establishing compliance with hip protectors, if their use is contemplated in the nursing home setting. Clearly, more research on effective strategies to reduce hip fractures in LTC is needed, including more studies on pharmacologic treatments, exercise, behavioural, or multi-modal strategies, and hip protectors.

## Supporting Information

Checklist S1 Prisma Checklist
(0.07 MB DOC)

Appendix S1 (0.04 MB DOC)
