# Supplementary material for: A Scoping Review of Strategies for the Prevention of Hip Fracture in Elderly Nursing Home Residents
Source: PLoS One. 2010 Mar 3;5(3):e9515. doi: 10.1371/journal.pone.0009515 (PMC2831075; doi:10.1371/journal.pone.0009515)
Supplement: Appendix S1 — (0.04 MB DOC) [file pone.0009515.s002.doc]

**S1 Appendix. Details of the electronic searches**

The following respective electronic searches for citations were conducted: a) vitamin D and calcium - Medline, EMBASE, CINAHL, AMED (Allied and Complementary Medicine Database), and AgeLine were searched on February 4, 2008 and updated January 15, 2009, b) non-hormonal pharmacologic therapies for osteoporosis - Medline, EMBASE, CINAHL, HealthStar, and AgeLine, were searched on December 26, 2007 with DARE on January 11, 2008, and updated on January 16, 2009, c) hormonal therapies - Medline, EMBASE, CINAHL, and AgeLine were searched on September 26, 2008, d) oral or parenteral complementary or alternative medicines - Medline, EMBASE, CINAHL, AMED (Allied and Complementary Medicine Database), and AgeLine were searched on May 2, 2008, e) exercise, behavioral interventions, or multimodal interventions – Medline, EMBASE, and CINAHL were searched on March 11, 2008 and May 8, 2009, and f) hip protectors - Medline, EMBASE, and Medline-in-Process and other non-indexed citations, were searched on April 22, 2009, whereas CINAHL was updated on August 22, 2008 (update of a prior search detailed in reference 22). More details about search terms are available upon request from the corresponding author. An example of a search undertaken in Ovid Medline for calcium and vitamin D (or vitamin D analogues) is shown below:

Database: Ovid MEDLINE(R) <1950 to January Week 4 2008>

1 calcium/ (205875)

2 calcium.tw. (217576)

3 exp Vitamin D/ (30316)

4 (alfacalcidiol or alfacalcidol).tw. (243)

5 rocaltrol.tw. (45)

6 Calcitriol/ (10824)

7 calcitriol.tw. (2586)

8 (1,25-dihydroxyvitamin adj D3).tw. (3894)

9 exp Ergocalciferols/ (2481)

10 ergocalciferol?.tw. (231)

11 ((vitamin adj D3) or "vitamin D 3").tw. (5864)

12 exp Cholecalciferol/ (18285)

13 (cholecalciferol? or colecalciferol?).tw. (950)

14 dihydrotachysterol.tw. (270)

15 (25-hydroxyvitamin adj D3).tw. (1214)

16 Calcifediol/ (2190)

17 calcifediol.tw. (40)

18 "25(OH)D3".tw. (817)

19 "1-alpha(OH)D3".tw. (260)

20 (1-alphahyroxyvitamin adj D).tw. (0)

21 Cod Liver Oil/ (313)

22 "cod liver oil?".tw. (475)

23 exp Fish Oils/ (11566)

24 (fish adj oil?).tw. (4696)

25 "fish liver oil?".tw. (46)

26 1-hydroxycholecalciferol.nm. (748)

27 1-hydroxycholecalciferol.tw. (2)

28 or/1-27 (349568)

29 exp Hip Fractures/ (12200)

30 fracture$.tw. (110443)

31 or/29-30 (112730)

32 Homes For The Aged/ or exp Nursing Homes/ (29126)

33 Long-Term Care/ (17420)

34 Housing for the Elderly/ (1185)

35 exp Aged/ (1662473)

36 (nursing adj (home? or house or facilit$ or residence)).tw. (16450)

37 ((long term or long-term or longterm) adj care).tw. (9544)

38 "home for the aged".tw. (296)

39 (old adj age adj (home? or house or facilit$ or residence)).tw. (211)

40 (retirement adj (home? or house or facilit$ or residence)).tw. (154)

41 (((senior adj citizen?) or senior?) adj (home? or house or facilit$ or residence)).tw. (469)

42 (institution$ adj ((older adj adult?) or elderly or patient? or aged)).tw. (2333)

43 (residential adj (care or accommodation?)).tw. and (elderly or aged or senior? or "old age" or "older adult").mp. (762)

44 or/32-43 (1687020)

45 28 and 31 and 44 (2018)

46 randomized controlled trial.pt. (246660)

47 controlled clinical trial.pt. (76052)

48 randomized controlled trials/ (51847)

49 random allocation/ (59418)

50 double blind method/ (94259)

51 single blind method/ (11548)

52 clinical trial.pt. (439482)

53 clinical trial/ (438541)

54 (clin$ adj25 trial$).ti,ab. (139080)

55 ((singl$ or double$ or trebl$ or tripl$) adj25 (blind$ or mask$)).ti,ab. (93467)

56 placebos/ (26524)

57 placebo$.ti,ab. (106301)

58 random$.ti,ab. (393128)

59 research design/ (50559)

60 comparative study.pt. (1361761)

61 exp evaluation studies/ (96522)

62 follow up studies/ (352117)

63 prospective studies/ (233278)

64 (control$ or prospectiv$ or volunteer$).ti,ab. (1863893)

65 or/46-64 (3672787)

66 animals/ not human/ (3141649)

67 65 not 66 (2843961)

68 randomized controlled trial.pt. (246660)

69 random$.tw. (393128)

70 control$.tw. (1602464)

71 intervention?.tw. (261640)

72 evaluat$.tw. (1270035)

73 or/68-72 (2994054)

74 animals/ not (animals/ and humans/) (3141649)

75 73 not 74 (2318596)

76 search$.tw. (115491)

77 meta-analysis.mp,pt. (28255)

78 review.pt. (1333714)

79 di.xs. (3180402)

80 associated.tw. (1318713)

81 or/76-80 (5069761)

82 67 or 75 or 81 (7069142)

83 45 and 82 (1783)
